# Hybrid Shape Memory Alloy-Based Nanomechanical Resonators for Ultrathin Film Elastic Properties Determination and Heavy Mass Spectrometry

**DOI:** 10.3390/ma12213593

**Published:** 2019-10-31

**Authors:** Ivo Stachiv, Lifeng Gan

**Affiliations:** 1 Institute of Physics, Czech Academy of Sciences, Na Slovance 2, 18221 Prague, Czech Republic; 2 School of Sciences, Harbin Institute of Technology, Shenzhen, Shenzhen 518055, Guangdong, China; lifeng.gan@outlook.com; 3 Drážní Revize s.r.o., Místecká 1120/103, 70300 Ostrava—Vitkovice, Czech Republic

**Keywords:** shape memory alloys, phase transformation, mass spectrometry, Young’s modulus, nanomechanics, resonator, *Q*-factor

## Abstract

Micro-/nanomechanical resonators are often used in material science to measure the elastic properties of ultrathin films or mass spectrometry to estimate the mass of various chemical and biological molecules. Measurements with these sensors utilize changes in the resonant frequency of the resonator exposed to an investigated quantity. Their sensitivities are, therefore, determined by the resonant frequency. The higher resonant frequency and, correspondingly, higher quality factor (*Q*-factor) yield higher sensitivity. In solution, the resonant frequency (*Q*-factor) decreases causing a significant lowering of the achievable sensitivity. Hence, the nanomechanical resonator-based sensors mainly operate in a vacuum. Identification by nanomechanical resonator also requires an additional reference measurement on the identical unloaded resonator making experiments, due to limiting achievable accuracies in current nanofabrication processes, yet challenging. In addition, the mass spectrometry by nanomechanical resonator can be routinely performed for light analytes (i.e., analyte is modelled as a point particle). For heavy analytes such as bacteria clumps neglecting their stiffness result in a significant underestimation of determined mass values. In this work, we demonstrate the extraordinary capability of hybrid shape memory alloy (SMA)-based nanomechanical resonators to i) notably tune the resonant frequencies and improve *Q*-factor of the resonator immersed in fluid, ii) determine the Young’s (shear) modulus of prepared ultrathin film only from frequency response of the resonator with sputtered film, and iii) perform heavy analyte mass spectrometry by monitoring shift in frequency of just a single vibrational mode. The procedures required to estimate the Young’s (shear) modulus of ultrathin film and the heavy analyte mass from observed changes in the resonant frequency caused by a phase transformation in SMA are developed and, afterward, validated using numerical simulations. The present results demonstrate the outstanding potential and capability of high frequency operating hybrid SMA-based nanomechanical resonators in sensing applications that can be rarely achieved by current nanomechanical resonator-based sensors.

## 1. Introduction

Because of their small mass, high operating frequencies and the extraordinary sensitivity to external stimuli, micro-/nanomechanical resonators are used in various sensing devices including infrared thermal [1], fluid viscosity [2,3], force [4,5] and mass sensors [6,7,8,9,10]. For example, if analyte is adsorbed on the resonator surface, it shifts the resonant frequencies to lower values. Then, from a known functional dependency of the resonant frequency on the analyte mass and position of attachment, the mass of analyte can be estimated [9,10]. Importantly, this procedure for mass determination does not account for effect of the analyte stiffness on the resonant frequency, therefore the one is strictly valid to a light analyte mass (i.e., analyte mass is much smaller than the resonator mass). For heavy analytes, such as large biological or bacteria clumps, their stiffness must be accounted making heavy mass spectrometry by current nanomechanical resonators highly challenging task [11].

The relatively simple manufacturing, handling, actuation and detection via an opto-mechanical coupling made the micro-/nanomechanical resonators also suitable for non-destructive material characterization of thin films [12,13,14,15,16,17,18]. Recently, the nanofabrication and integrated circuit technologies continue to scale towards the ultimate limit of individual atoms or molecules enabling design the advanced nanoelectromechanical systems (NEMS). These systems have functional length scale ranging from a few nm to several hundreds of nm, thus they can be considered in applications not yet feasible by current microscale devices [19,20,21]. To prevent the mechanical failure of prepared NEMS, their elastic properties must be known. Determination of the nanomaterials’ elastic properties is difficult because for nanosized samples not only the Young’s (shear) modulus but also density and other material properties, due to a large surface-to-volume ratio, differ notably from known bulk values. As such, multiple experimental techniques including the nanoindentation [22], X-ray diffraction [23] and the resonant methods [12,13,14,15,16,17,18] were developed to determine the elastic properties in nanoscale. Among them, just the resonant methods can be integrated in situ into the nanomaterials deposition systems [24]. Nevertheless, the material characterization by resonant methods requires the reference measurement on the identical uncoated resonator complicating experiments (i.e., the limiting achievable accuracy in nanofabrication processes and the necessity of precisely controlling the experimental conditions in nanoscale). We note that the sensitivity of nanomechanical resonator-based sensors is related to the resonant frequency [25]. The higher resonant frequency and quality factor (*Q*-factor) yield higher sensitivity. When resonator operates in air or fluid, dissipative forces caused by surrounding medium dominate shifting the resonant frequencies (*Q*-factors) to the lower values. Thus, most currently developed nanomechanical resonator-based sensors operate in vacuum.

Shape memory alloys (SMAs) pose unique functional properties like shape memory effect and superelasticity [26]. These effects are linked with temperature and/or stress driven a diffusionless martensitic transformation of SMA. Moreover, the high energy density makes SMA highly attractive for microactuation purposes [27,28]. The SMA-based microactuators are usually set into a periodic motion by a heat induced phase transformation. These actuators are, therefore, with a relatively low actuating speed of a few kHz [28] caused by the necessity of cooling the SMA components. Recently, hybrid SMA-based nanomechanical resonators made of an elastic substrate and NiTi in form of thin film were proposed [29,30]. These hybrid resonators were proven to operate at high resonant frequency ranges (i.e., up to tens of MHz) that can be significantly tuned up and down making them suitable for applications, such as radio frequency filters, infrared and temperature sensors.

In this paper, we first expand previous work [29], where the high frequency SMA resonators vibrating in vacuum were investigated, to account for fluid dissipative forces and, consequently, to reveal impact of a phase transformation of NiTi film on the achievable resonant frequency and *Q*-factor values and, correspondingly, sensitivity of the nanomechanical resonator operating in air or viscous fluid. Secondly, we show that variable effective elasticity of hybrid SMA-based nanomechanical resonators enable determination of the Young’s and shear moduli of prepared ultrathin film from only measured changes in the resonant frequency of resonator with sputtered film, and the heavy analyte mass from detected frequency shift of just a single vibrational mode. The explicit analytical formulae for the frequency response of the hybrid SMA-based resonators immersed in viscous fluid are given. Procedures of the ultrathin film elastic (shear) modulus and analyte mass determinations by means of hybrid SMA-based nanomechanical resonators are developed and their validity are reinforced using numerical simulations.

## 2. Theory

We begin with theoretical models that enable accurately predict spectrum of the flexural and torsional resonant frequencies and *Q*-factors of the hybrid SMA-based nanomechanical resonator operating in air and/or viscous fluid. These resonators consist of an elastic substrate and one or multiple material layer films including phase changeable SMA (NiTi) film, protective coating(s) and active materials layer films, etc.

### 2.1. Flexural Oscillations of Hybrid SMA (NiTi) Based Nanomechanical Resonator Immersed in Viscous Fluid

**Figure 1:**
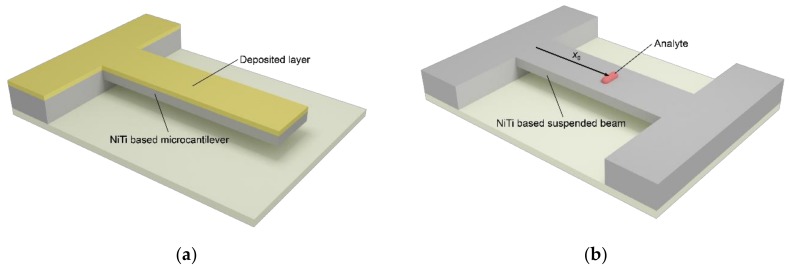
Sketch of hybrid SMA (NiTi)-based nanomechanical resonator in (**a**) cantilever configuration and with deposited ultrathin film; and (**b**) suspended configuration and with analyte mass.

The SMA (NiTi) film generates the interlayer in-plane stress over the entire resonator surface [29,30]. This stress is either tensile or compressive depending on the resonator dimensions and material properties, preparation processes and the final resonator configuration [31]. Thus, the general form of governing equation for the flexural motion of hybrid SMA (NiTi)-based nanomechanical resonator immersed in viscous fluid is given by

(1) $$A_{0}\frac{\partial^{2}u\left(x,t\right)}{\partial t^{2}}+D_{F}\frac{\partial^{4}u\left(x,t\right)}{\partial x^{4}}\mp F_{\sigma }\left(x,t\right)\frac{\partial^{2}u\left(x,t\right)}{\partial x^{2}}=F_{\mathrm{drive}}\left(x,t\right)+F_{\mathrm{hydro}}\left(x,t\right)$$

where *u*(*x*,*t*) is the resonator deflection, $A_{0}=\sum_{i=1}^{N}\rho_{i}S_{i}$ is the linear density, $D_{F}=\sum_{i=1}^{N}E_{i}\int_{\prod i}^{}u^{*2}dS-\frac{{\left(\sum_{i=1}^{N}E_{i}\int_{\prod i}^{}u^{*}dS\right)}^{2}}{\sum_{i=1}^{N}E_{i}S_{i}}\;$ is the flexural rigidity, *N* is the number of material layers, *S* is the resonator cross-sectional area, *ρi* and *Ei* are the density and the Young’s modulus of *i*-th material layer, u* is the local coordinate in the lateral direction, Π*i* is the *i*-th region of resonator cross-section [32], *Ti* is the thickness of *i*-th material layer, *W* is the resonator width, $F_{\sigma }\left(x,t\right)\approx W\sum_{i=1}^{N}\sigma_{i}T_{i}$ is the average interlayer force per unit length mainly originating from a phase transforming SMA (NiTi) film [29,30,31], σ is the average axial stress induced in *i*-th material layer, + (−) sign stands for the compressive (tensile) value of the interlayer force, *F*_drive_(*x*,*t*) is an arbitrary form of the external driving force per unit length that actuates the resonator, *F*_hydro_(*x*,*t*) is the hydrodynamic load due to surrounding fluid. For cantilever configuration (Figure 1a) of the SMA resonator the following boundary conditions are imposed:

(2) $$u\left(0,t\right)=0,\;\frac{\partial u\left(0,t\right)}{\partial x}=0,\;\frac{\partial^{2}u\left(L,t\right)}{\partial x^{2}}=0,\;D_{F}\frac{\partial^{3}u\left(L,t\right)}{\partial x^{3}}=F_{\sigma }\left(L,t\right)\frac{\partial u\left(L,t\right)}{\partial x}.\;$$

while for SMA resonator in suspended configuration (see Figure 1b) the boundary conditions are

(3) $$u\left(0,t\right)=0,\;\frac{\partial u\left(0,t\right)}{\partial x}=0,\;u\left(L,t\right)=0,\;\frac{\partial u\left(L,t\right)}{\partial x}=0$$

where *L* is the resonator length.

We are interested in analyzing the impact of a phase transformation in SMA film on frequency response of the nanomechanical resonator immersed in fluid; therefore, the solution is going to be obtained in the frequency domain. Solving the Fourier transformed continuity, ∇·**u** = 0, and the Navier-Stokes, *j*ω*ρ*_fluid_ = ∇*p*, equations for an incompressible fluid, where **u** is the velocity field, *ρ*_fluid_ is the fluid density, *p* is the hydrodynamic pressure, yields the required general form of the hydrodynamic load [33]:

(4) $$F_{\mathrm{hydro}}\left(x|\omega \right)=\frac{\pi }{4}\rho_{\mathrm{fluid}}\omega^{2}W^{2}{Г}_{F}\left(\omega \right)U\left(x|\omega \right)$$

Substituting Equation (4) in the scaled with respect to the resonator length the Fourier transformed Equation (1) and rearranging terms, we obtain

(5) $$\frac{d^{4}U\left(x|\omega \right)}{dx^{4}}\mp b^{2}\frac{d^{2}U\left(x|\omega \right)}{dx^{2}}-{\left(\gamma_{\left(n\right)}^{2}\frac{\omega }{\omega_{\mathrm{vac}\_F}^{\left(n\right)}}\right)}^{2}\left[1+\frac{\pi }{4}\frac{\rho_{\mathrm{fluid}}W^{2}}{A_{0}}{Г}_{F}\left(\omega \right)\right]U\left(x|\omega \right)={\tilde{F}}_{\mathrm{drive}}\left(x|\omega \right)$$

where *U*(*x*|*w*) is the resonator dynamic deflection in the complex space, *b*=*L*$\sqrt{F_{\sigma }/D_{F}}$ is the dimensionless tension parameter $\omega_{\mathrm{vac}\_F}^{\left(n\right)}={\left(\frac{\gamma_{\left(n\right)}}{L}\right)}^{2}\sqrt{D_{F}/A_{0}}$ is the angular flexural resonant frequency of the *n*-th nanomechanical resonator flexural vibrational mode in vacuum obtained by solving Equation (5) for ${\tilde{F}}_{\mathrm{drive}}\left(x|\omega \right)=F_{\mathrm{hydro}}\left(x|\omega \right)=0$, and *γ*_(__*n*)_ is the *n*-th positive root of the appropriate transcendental equation. For cantilever configuration of the SMA-based nanomechanical resonator this transcendental equation is given by

(6) $$\left(1+\frac{b^{4}}{2q_{1}^{2}q_{2}^{2}}\right)\cosh q_{i}\cos q_{k}+1\mp \frac{b^{2}}{2q_{1}q_{2}}\sinh q_{i}\sin q_{k}=0$$

and, similarly, for suspended SMA resonator the one reads

(7) $$\cosh q_{i}\cos q_{k}-1\mp \frac{b^{2}}{2q_{1}q_{2}}\sinh q_{i}\sin q_{k}=0$$

where $q_{1,2}=\sqrt{\pm \tilde{b}+\sqrt{{\tilde{b}}^{2}+\gamma^{4}}}$, $\tilde{b}=b/2$, *i* = 1(2) and *k* = 2(1) stand for tensile/compressive stress.

The general solution for flexural deflection of the SMA-based nanomechanical resonator can be obtained using the eigenfunction expansion method. We seek a solution in the following form

(8) $$U\left(x|\omega \right)=\sum_{n=1}^{n}B_{F\left(n\right)}\left(\omega \right)\theta_{F\left(n\right)}\left(x\right)$$

where *B*_*F*(*n*)_(*w*) is the frequency dependent parameter that must be determined, and *θ*_*F*(*n*)_(*x*) is the mode shape, which for cantilever configuration of the SMA resonator reads

(9) $$\theta_{F\left(n\right)}\left(x\right)=\cos h\left(q_{1\left(n\right)}x\right)-\cos\left(q_{2\left(n\right)}x\right)-\left[\frac{\cos h\left(q_{1\left(n\right)}\right)+{\left(\frac{q_{2\left(n\right)}}{q_{1\left(n\right)}}\right)}^{2}\cos\left(q_{2\left(n\right)}\right)}{\sin h\left(q_{1\left(n\right)}\right)+\frac{q_{2\left(n\right)}}{q_{1\left(n\right)}}\sin\left(q_{2\left(n\right)}\right)}\right]\left[\sin h\left(q_{1\left(n\right)}x\right)-\frac{q_{1\left(n\right)}}{q_{2\left(n\right)}}\sin\left(q_{2\left(n\right)}x\right)\right]$$

and for suspended one the mode shape is given by

(10) $$\theta_{F\left(n\right)}\left(x\right)=\cos h\left(q_{1\left(n\right)}x\right)-\cos\left(q_{2\left(n\right)}x\right)-\left[\frac{\cos h\left(q_{1\left(n\right)}\right)-\cos\left(q_{2\left(n\right)}\right)}{\sin h\left(q_{1\left(n\right)}\right)-\frac{q_{1\left(n\right)}}{q_{2\left(n\right)}}\sin\left(q_{2\left(n\right)}\right)}\right]\left[\sin h\left(q_{1\left(n\right)}x\right)-\frac{q_{1\left(n\right)}}{q_{2\left(n\right)}}\sin\left(q_{2\left(n\right)}x\right)\right]$$

Substituting Equation (8) into Equation (5) and using the orthonormal properties of $\theta_{F\left(n\right)}\left(x\right)$, the frequency dependent parameter $B_{F\left(n\right)}\left(\omega \right)$ can be calculated from the following expression

(11) $$B_{F\left(n\right)}\left(\omega \right)=\frac{\int_{0}^{L}{\tilde{F}}_{\mathrm{dr}}\left(\bar{x}|\omega \right)\theta_{F\left(n\right)}\left(\bar{x}\right)d\bar{x}}{\gamma_{\left(n\right)}^{4}\mp {\left(b\gamma_{\left(n\right)}\right)}^{2}-{\left(\gamma_{\left(n\right)}^{2}\frac{\omega }{\omega_{v_{F}}^{\left(n\right)}}\right)}^{2}\left[1+\frac{\pi }{4}\frac{\rho_{\mathrm{fluid}}W^{2}}{A_{0}}\Gamma_{F}\left(\omega \right)\right]}$$

It is a well-known fact the quality factor must be sufficiently high (i.e., *Q*^(*n*)^ >> 1). Just in this case the resonant frequency of the *n*-th vibrational mode of the nanomechanical resonator can be correctly identified enabling measurement of the desired quantity [25]. For *Q*^(*n*)^ >> 1, the analogy with a simple harmonic oscillator can be used. Then, the hydrodynamic function in a vicinity of the resonance peaks can be expressed as a combination of the fluid dissipative, $\Gamma_{F\_im}\left(\omega_{F}^{\left(n\right)}\right)$, and added mass, $\Gamma_{F\_r}\left(\omega_{F}^{\left(n\right)}\right)$, effects as $\Gamma_{F}\left(\omega \right)=\Gamma_{F\_r}\left(\omega_{F}^{\left(n\right)}\right)+j\Gamma_{F\_im}\left(\omega_{F}^{\left(n\right)}\right)$ [6,33]. Consequently, for $\Gamma_{F\_r}\left(\omega_{F}^{\left(n\right)}\right)\gg \Gamma_{F\_im}\left(\omega_{F}^{\left(n\right)}\right)$ the flexural resonant frequency and *Q*-factor of the *n*-th vibrational mode of the hybrid SMA-based nanomechanical resonator immersed in air or viscous fluid can be accurately predicted by

(12) $$f_{F}^{\left(n\right)}=\frac{f_{\mathrm{vac}\_F}^{\left(n\right)}}{\sqrt{1+\frac{\pi }{4}\frac{\rho_{\mathrm{fluid}}W^{2}}{A_{0}}\Gamma_{F\_r}\left(\omega_{F}^{\left(n\right)}\right)}}$$

(13) $$Q_{F}^{\left(n\right)}=\frac{\frac{4}{\pi }\frac{A_{0}}{\rho_{\mathrm{fluid}}W^{2}}+\Gamma_{F\_r}\left(\omega_{F}^{\left(n\right)}\right)}{\Gamma_{F\_im}\left(\omega_{F}^{\left(n\right)}\right)}$$

where *f* = ω/(2π).

### 2.2. Torsional Oscillations of Hybrid SMA (NiTi) Based Nanomechanical Resonator Immersed in Viscous Fluid

In contrast to flexural oscillations, where both the variable flexural rigidity and interlayer stress have a direct impact on the frequency response, the torsional modes are affected only by the SMA (NiTi) film induced variable torsional modulus of elasticity. Using the membrane analogy of Prandtl [34], the general equation of motion for torsional oscillations of the SMA-based nanomechanical resonator can be written as

(14) $$D_{Tr}\frac{\partial^{2}\phi \left(x,t\right)}{\partial x^{2}}-A_{1}\frac{\partial^{2}\phi \left(x,t\right)}{\partial t^{2}}=M_{\mathrm{drive}}\left(x,t\right)+M_{\mathrm{hydro}}\left(x,t\right)$$

where *ϕ*(*x*, *t*) is the deflection angle about the cantilever major axis, *M*_drive_(*x*, *t*) and *M*_hydro_(*x*, *t*) are the external driving torque per unit length and the hydrodynamic torque per unit length [35], $D_{Tr}=\overset{N}{\underset{i=1}{\sum }}G_{i}\int_{\Pi_{i}}^{}\phi^{*2}\mathrm{dS}-\frac{{\left(\sum_{i=1}^{N}G_{i}\int_{\Pi_{i}}^{}\phi^{*}\mathrm{dS}\right)}^{2}}{\sum_{i=1}^{n}G_{i}S_{i}}$, $A_{1}=\overset{N}{\underset{i=1}{\sum }}\rho_{i}I_{Pi}$, *I*_P*i*_ is the resonator polar moment of inertia, *G* is the shear modulus and ϕ* is the local coordinate. The torsional oscillations are realized just for the resonator in cantilever configuration, of which the boundary conditions are

(15) $$\phi \left(0,t\right)=0,\frac{\partial \phi \left(L,t\right)}{\partial x}=0$$

Due to apparent similarities with the flexural motion, the solution for torsional oscillations of the hybrid SMA (NiTi)-based nanomechanical resonator can be obtained in the same manner as done previously for flexural one. Plugging the general form of the hydrodynamic torque $M_{\mathrm{hydro}}\left(x|\omega \right)=-\frac{\pi }{8}\rho_{\mathrm{air}}\omega^{2}W^{4}\Gamma_{T}\left(\omega \right)\Phi \left(x|\omega \right)$ [35] into scaled with respect to the cantilever length the Fourier-transformed Equation (14) and rearranging terms yields

(16) $$\frac{d^{2}\Phi \left(x|\omega \right)}{dx^{2}}-{\left(\lambda_{n}\;\frac{\omega }{\omega_{\mathrm{vac}\_T}^{\left(n\right)}}\right)}^{2}\left[1+\frac{\pi }{8}\frac{\rho_{\mathrm{fluid}}W^{4}}{A_{1}}\Gamma_{T}\left(\omega \right)\right]\Phi \left(x|\omega \right)={\tilde{M}}_{\mathrm{drive}}\left(x|\omega \right)$$

where $\;\Phi \left(x|\omega \right)$ is the resonator deflection angle in complex space, ${\tilde{M}}_{\mathrm{drive}}\left(x|\omega \right)=\;\frac{M_{\mathrm{drive}}\left(x|\omega \right)L^{2}}{D_{Tr}}$, $\omega_{\mathrm{vac}\_T}^{\left(n\right)}=\frac{\lambda_{n}}{L}\;\sqrt{\frac{D_{Tr}}{A_{1}}}$ are the angular torsional resonant frequencies of the cantilever resonator vibrating in vacuum, and *λ*_n_ = *π*(2*n* – 1)/2, *n* = 1, 2, 3,… are the cantilever resonator dimensionless torsional resonant frequencies. The solution for the torsional frequency response of the SMA-based cantilever resonator immersed in viscous fluid and driven by an arbitrary moment is sought in form

(17) $$\Phi \left(x|\omega \right)=\overset{\infty }{\underset{n=1}{\sum }}C_{Tr\left(n\right)}\left(\omega \right)\theta_{Tr\left(n\right)}\left(x\right)$$

where $\theta_{Tr\left(n\right)}\left(x\right)=\sin\left[\left(2n-1\right)\pi x/2\right]$ and $C_{Tr\left(n\right)}\left(\omega \right)$ is determined from

(18) $$C_{Tr\left(n\right)}\left(\omega \right)=\frac{\int_{0}^{L}{\tilde{M}}_{dr}\left(\bar{x}|\omega \right)\theta_{Tr\left(n\right)}\left(\bar{x}\right)d\bar{x}}{\lambda_{\left(n\right)}^{2}{\left(\frac{\omega }{\omega_{vac\_T}^{\left(n\right)}}\right)}^{2}\left[1+\frac{\pi }{8}\frac{\rho_{fluid}W^{4}}{A_{1}}\Gamma_{T}\left(\omega \right)\right]}$$

Using the same argument as for the flexural oscillations, the desired expressions enabling accurately predicting the torsional resonant frequency and *Q*-factor of the *n*-th vibrational mode of the hybrid SMA-based nanomechanical resonator operating in viscous fluid are

(19) $$\omega_{Tr}^{\left(n\right)}=\frac{\omega_{vac\_Tr}^{\left(n\right)}}{\sqrt{1+\frac{\pi }{8}\frac{\rho_{fluid}W^{4}}{A_{1}}\Gamma_{T\_r}\left(\omega_{T}^{\left(n\right)}\right)}}$$

(20) $$Q_{Tr}^{\left(n\right)}=\frac{\frac{8}{\pi }\frac{A_{1}}{\rho_{fluid}W^{4}}+\Gamma_{T\_r}\left(\omega_{Tr}^{\left(n\right)}\right)}{\Gamma_{T\_im}\left(\omega_{Tr}^{\left(n\right)}\right)}$$

where $\Gamma_{T\_r}\left(\omega_{Tr}^{\left(n\right)}\right)$ and $\Gamma_{T\_im}\left(\omega_{Tr}^{\left(n\right)}\right)$ are known real and imaginary components of the hydrodynamic function obtained by solving the flow generated by resonator executing torsional oscillations [35].

## 3. Results and Discussion

### 3.1. Impact of Phase Transformation in SMA (NiTi) Film on Resonant Frequencies and Q-factor of Nanomechanical Resonator Operating in Viscous Fluid

To simplify the following analysis, we assume without a loss of generality that hybrid SMA-based nanomechanical resonator consists of an elastic substrate made of either Si (*ρ* = 2.33 g/cm^3^, *E* = 169 GPa), SiO_2_ (*ρ* = 2.65 g/cm^3^, *E* = 69 GPa) or SU-8 photoresist (*ρ* = 1.19 g/cm^3^, *E* = 4 GPa), and sputtered NiTi film. The flexural rigidity of two layered structure is

(21) $$D_{F}=\frac{1}{12}WT_{1}^{3}E_{1}r\left(\xi ,\eta \right)$$

where *r*(*ξ*,*η*) = [*ξ*^2^*η*^4^ + 4*ξη*(1+ 1.5*η* + *η*^2^) + 1]/(1 + *ξη*), *ξ* = *E*_2_/*E*_1_, *η* = *T*_2_/*T*_1_, subscripts 1 and 2 stand for the substrate and NiTi film, respectively. Introducing the dimensionless thickness parameters *h*_1_ = *T*_1_/(*T*_1_ + *T*_2_) and *h*_2_ = *T*_2_/(*T*_1_ + *T*_2_), and accounting for a moment of inertia in form (1/12)*W*(*T*_1_ + *T*_2_)^3^ the effective Young’s modulus of a SMA (NiTi)-based nanomechanical resonator can be expressed as

(22) $$E_{\mathrm{eff}}=\frac{E_{1}^{2}h_{1}^{4}+E_{2}^{2}h_{2}^{4}+4E_{1}E_{2}h_{1}^{3}h_{2}+6E_{1}E_{2}h_{1}^{2}h_{2}^{2}+4E_{1}E_{2}h_{1}h_{2}^{3}}{E_{1}h_{1}+E_{2}h_{2}}.$$

**Figure 2:**
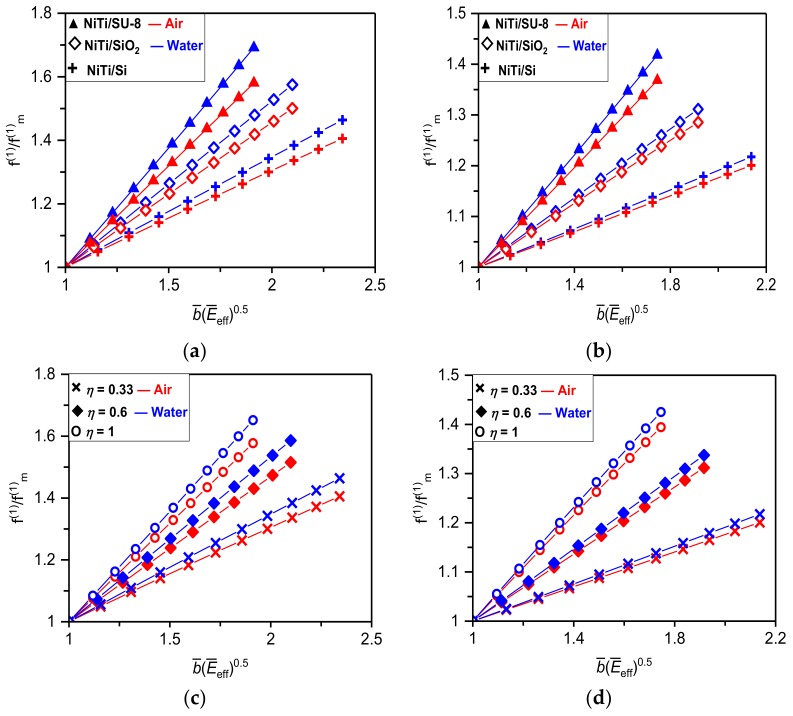
Changes in the fundamental flexural resonant frequency of NiTi/SU-8, NiTi/SiO_2_ and NiTi/Si nanomechanical resonators of *L* = 10 μm and thickness ratio *η* = 0.33 in (**a**) cantilever and (**b**) suspended configuration immersed in air and water. Dependencies of changes in the fundamental flexural resonant frequency of NiTi/Si nanomechanical resonator in (**c**) cantilever and (**d**) suspended configuration on different NiTi film thickness ratios *η* = 0.33, 0.6 and 1.

**Figure 3:**
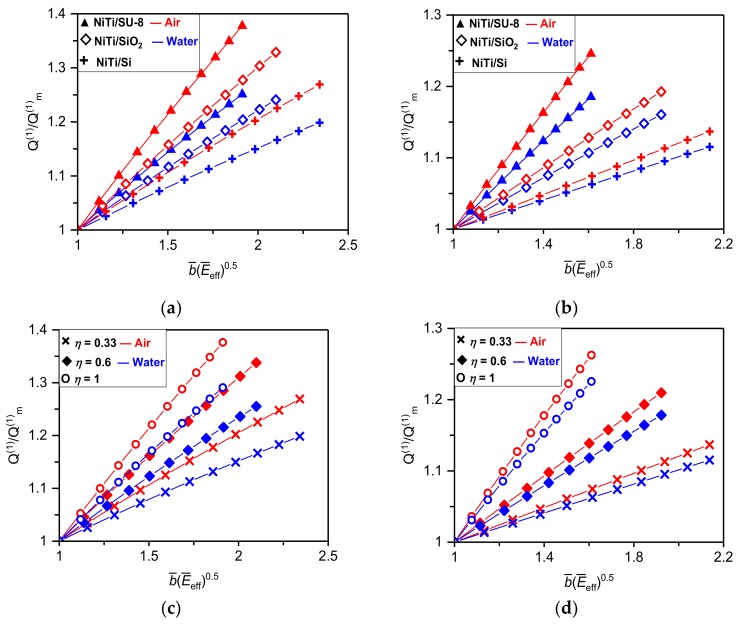
Changes in the *Q*-factor of the fundamental flexural mode of NiTi/SU-8, NiTi/SiO_2_ and NiTi/Si nanomechanical resonators of *L* = 10 μm and thickness ratio *η* = 0.33 in (**a**) cantilever and (**b**) suspended configuration immersed in air and water. Dependencies of changes in *Q*-factor of the fundamental flexural mode of NiTi/Si nanomechanical resonator in (**c**) cantilever and (**d**) suspended configuration on different NiTi film thickness ratios *η* = 0.33, 0.6 and 1.

We note that the Young’s and shear moduli are interrelated to each other by *E* = 2*G*(1 + *ν*), where *ν* is the Poisson’s ratio. The phase transformation in sputtered NiTi film is accompanied with a significant change in the resonator effective elastic properties [29,30,31]. Since heating of NiTi is essentially faster than cooling, therefore the martensite phase of NiTi is considered to be an initial state of the SMA (NiTi)-based nanomechanical resonators. The Young’s modulus of low temperature martensite phase ranges from 25 to 40 GPa, while the high temperature austenite phase has the Young’s modulus from 60 to 83 GPa. Importantly, during preparation of the crystalline NiTi film either a tensile or compressive interlayer stress of tens kPa to hundreds of MPa depending on multiple physical and material effects is generated within designed structure [31]. During heating, the interlayer stress usually increases to values up to several hundreds of MPa. It immediately implies that the flexural resonant frequencies and, corresponding, *Q*-factor values depend on the temperature-dependent dimensionless modulus parameter ξ and tension parameter *b*. Consequently, changes in the flexural resonant frequency and *Q*-factor can be fully characterized by the following two dimensionless parameters:

(23) $${\bar{E}}_{\mathrm{eff}}=\frac{E_{\mathrm{eff}}}{E_{\mathrm{eff}\_m}}=\frac{r\left(\xi ,\eta \right)}{r\left(\xi_{m},\eta \right)},\;\bar{b}=\frac{b}{b_{m}}=\sqrt{\frac{F_{\sigma }}{F_{\sigma \_m}}\frac{1}{{\bar{E}}_{\mathrm{eff}}}}$$

where subscript *m* stands for the martensite phase. The first parameter ${\bar{E}}_{\mathrm{eff}}$ represents importance of changes in the Young’s modulus of NiTi film on the resonator effective stiffness. During a phase transformation of NiTi from martensite to austenite, ${\bar{E}}_{\mathrm{eff}}$ increases indicating resonator stiffening caused by an increase of the NiTi film Young’s modulus. The second parameter $\bar{b}$ is ratio of the axial load to the effective Young’s modulus. This parameter describes impact of changes in the interlayer stress induced axial load during a phase transformation of NiTi. Dimensionless resonant frequencies obtained as positive roots of Equations (6) and (7) can be for an arbitrary value of interlayer stress accurately approximated using a polynomial function of order *N* [36]. Accounting for a polynomial dependency of $\gamma_{\left(n\right)}$ on *b*, Equations (12) and (13), and present discussion the relative changes in resonant frequency and *Q*-factor of the *n*-th vibrational mode of the hybrid SMA (NiTi)-based nanomechanical resonator caused by a phase transformation of NiTi film can be predicted by

(24) $$\frac{f_{F}^{\left(n\right)}}{f_{F\_m}^{\left(n\right)}}=\overset{N}{\underset{i=1}{\sum }}a_{i}{\bar{b}}^{i}\sqrt{{\bar{E}}_{\mathrm{eff}}^{2}\times \frac{\rho_{1}S_{1}+\rho_{2}S_{2}+\kappa_{F}\rho_{\mathrm{fluid}}W^{2}\Gamma_{F\_r}\left(\omega_{F\_m}^{\left(n\right)}\right)}{\rho_{1}S_{1}+\rho_{2}S_{2}+\kappa_{F}\rho_{\mathrm{fluid}}W^{2}\Gamma_{F\_r}\left(\omega_{F}^{\left(n\right)}\right)}}\approx \overset{N}{\underset{i=0}{\sum }}a_{i}{\bar{b}}^{i}{\bar{E}}_{\mathrm{eff}},$$

(25) $$\frac{Q_{F}^{\left(n\right)}}{Q_{F\_m}^{\left(n\right)}}=\frac{\rho_{1}S_{1}+\rho_{2}S_{2}+\kappa_{F}\rho_{\mathrm{fluid}}W^{2}\Gamma_{F\_r}\left(\omega_{F}^{\left(n\right)}\right)}{\rho_{1}S_{1}+\rho_{2}S_{2}+\kappa_{F}\rho_{\mathrm{fluid}}W^{2}\Gamma_{F\_r}\left(\omega_{F\_m}^{\left(n\right)}\right)}\times \frac{\Gamma_{F\_im}\left(\omega_{F\_m}^{\left(n\right)}\right)}{\Gamma_{F\_im}\left(\omega_{F}^{\left(n\right)}\right)}\approx \frac{\Gamma_{F\_im}\left(\omega_{F\_m}^{\left(n\right)}\right)}{\Gamma_{F\_im}\left(\omega_{F}^{\left(n\right)}\right)},$$

where $f_{F\_m}^{\left(n\right)}$ ($\omega_{F\_m}^{\left(n\right)}$) and $Q_{F\_m}^{\left(n\right)}$ are the simple (angular) resonant frequency and *Q*-factor of the *n*-th flexural vibrational mode of the resonator with NiTi film in a martensite phase, *a_i_* are the constants depending on the resonator configuration, i.e., cantilever or suspended, and the film/substrate thicknesses and elastic properties. Equations (24) and (25) show that effects of NiTi film on the resonant frequency and *Q*-factor differ essentially from each other. The variable elasticity and interlayer stress of NiTi have a direct impact on the resonator frequency yielding nearly a linear dependency of $f_{F}^{\left(n\right)}/f_{F\_m}^{\left(n\right)}$ on the dimensionless parameters represented by $\bar{b}\sqrt{{\bar{E}}_{\mathrm{eff}}}$ as shown in Figure 2. The *Q*-factor, however, depends only indirectly on the changeable properties of NiTi through a known frequency dependent imaginary component of the hydrodynamic function (i.e., a non-linear relationship between $\Gamma_{F\_im}$ and the frequency was found [33,35]). When frequency shifts to the higher values, $\Gamma_{F\_im}$ significantly decreases resulting in a corresponding non-linear increase of the resonator *Q*-factor as shown in Figure 3. Noticing that the real part of the hydrodynamic function, $\Gamma_{F\_r}$, has a negligibly small impact on frequency and *Q*-factor values [33,35,37].

Results given in Figure 2 and Figure 3 reveal that the higher relative changes in frequency or *Q*-factor can be achieved for cantilever configuration of the nanomechanical resonator consisting of NiTi film and the “soft” elastic substrate (e.g., for NiTi/SU-8 resonator the frequency tuning and *Q*-factor enhancement of ~70% and 48% are found). Furthermore, the higher relative frequency tuning can be realized for a resonator immersed in fluid (see Figure 2), while the larger *Q*-factor enhancement is achieved for resonator vibrating in air as evident from results given in Figure 3. Complementary results for the absolute frequency tuning and *Q*-factor enhancement caused by a phase transformation of NiTi film sputtered on the Si elastic substrate are summarized in Table 1 and Table 2. Obtained results show that even for the NiTi/Si resonator of *η* ≈ 0.17 the frequency tuning of 30% (35%) is observed for cantilever resonator immersed in air (DI water). Similarly, for suspended configuration of the identical resonator the frequency tuning in air and DI water are both close to 14%. Properties of the considered NiTi/Si resonator are: *E*_m_ = 30 GPa, *E*_a_ = 80 GPa (Austenite phase), *σ_m_* = 100 MPa, *σ_a_* = 300 MPa, *L* = 10 μm, W = 1 μm, *T*_1_ = 300 nm, and *T*_2_ = 50, 100 and 200 nm. The above presented findings would be of great importance in nanomechanical sensors applicable in biology or chemistry, where to preserve a native structure of cells or molecules all measurements must be carried out in gaseous or aqueous solutions.

**Figure 4:**
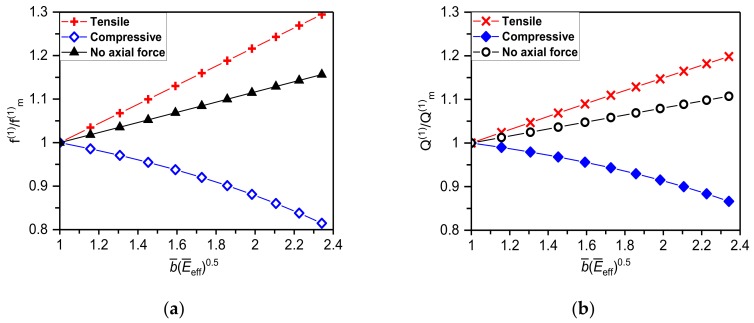
(**a**) Resonant frequency and (**b**) *Q*-factor enhancement of the fundamental flexural mode of the NiTi/Si nanocantilever of *L* = 6 μm, *T*_1_ = 300 and *T*_2_ = 100 nm caused by NiTi film phase transformation as a function of $\bar{b}\sqrt{{\bar{E}}_{\mathrm{eff}}}$.

In some cases, the interplay among different stresses (e.g., compressive stress induced by coefficients of thermal expansion mismatch combined with tensile stress due to a phase transformation in NiTi) can either generate the overall compressive interlayer stress acting upon the resonator or even do not generate any interlayer stress. Figure 4 shows dependencies of the fundamental flexural resonant frequency and, corresponding, *Q*-factor of the silicon cantilever nanomechanical resonator of 6 μm (*L*), 300 nm (*T*_1_), 100 nm (*T*_2_) on a phase transformation of NiTi film of 30 GPa (*E_m_*), 80 GPa (*E_a_*), 100 MPa (*σ_m_*) and 300 (*σ_a_*). As can be seen in Figure 4, tensile interlayer stress intensifies changes in the resonant frequencies and *Q*-factors. Compressive stress, in contrast, suppresses the effect of a phase transformation in NiTi yielding lowering of the resonant frequencies and *Q*-factors. It shall be pointed out that for no axial stress (i.e., σ = 0), an observed increase in frequency and *Q*-factor is caused just by the variable elasticity of NiTi film.

### 3.2. Young’s and Shear Moduli Determination Using SMA (NiTi) Based Nanomechanical Resonator

A hybrid SMA-based nanomechanical resonator is especially suitable for material characterization of prepared ultrathin film. During a phase transformation of NiTi, the resonator elastic properties are notably changed by the external stimulus, such as temperature. This unique characteristic of SMA resonator enables the Young’s modulus of ultrathin film being estimated from only ratio of two different measured resonant frequencies of the SMA resonator with sputtered film (e.g., frequency response in martensite (*f_m_*) and austenite (*f_a_*)). Using Equation (24), the frequency ratio yields

(26) $$\frac{f_{F\_a}^{\left(n\right)}}{f_{F\_m}^{\left(n\right)}}=\frac{\gamma_{a\_\left(n\right)}^{2}}{\gamma_{m\_\left(n\right)}^{2}}\sqrt{\frac{E_{a}\;r\left(\xi_{a},\eta \right)}{E_{m}\;r\left(\xi_{m},\eta \right)}}$$

where *ξ_m_* = *E*_2_/*E*_m_, *ξ_a_* = *E*_2_/*E*_a_, *r*(*ξ_a_*,*η*) = [*ξ_a_*^2^*η*^4^ + 4*ξ_a_η*(1 + 1.5*η* + *η*^2^) + 1]/(1 + *ξ_a_η*), *r*(*ξ_m_*,*η*) = [*ξ_a_*^2^*η*^4^ + 4*ξ_a_η*(1 + 1.5*η* + *η*^2^) + 1]/(1 + *ξ_a_η*), *E*_2_ is the Young’s modulus of sputtered film, *E*_m_ (*E_a_*), and *γ*_m_ (*γ*_a_) stand for known resonator “effective” Young’s modulus and the dimensionless resonant frequencies in marteniste (austenite) phase. For practical reasons, it is convenient to represent the dimensionless resonant frequencies of hybrid SMA-based nanomechanical resonator by a polynomial function. Following approach given in Ref. [36] and performing computations over a large number of interlayer stress values, elastic substrate material properties and resonator dimensions the appropriate polynomial dependencies of the first three flexural resonant frequencies of the SMA (NiTi)-based cantilever nanomechanical resonator on the generated tensile interlayer stress read

(27) $$\gamma_{\left(1\right)}^{2}=\left\{\begin{array}{c} \gamma_{B\left(1\right)}^{2}+0.6349b^{2}+0.00495b,\;b<0.5,\\ \gamma_{B\left(1\right)}^{2}+0.2375b^{2}+0.5547b,0.5\leq b\leq 3.2,\\ \gamma_{B\left(1\right)}^{2}-1.224+1.5342b,3.2<b, \end{array}\right\}$$

(28) $$\gamma_{\left(2\right)}^{2}=\left\{\begin{array}{c} \gamma_{B\left(2\right)}^{2}+0.7303b^{2}+0.0013b,\;b<0.5,\\ \gamma_{B\left(2\right)}^{2}+0.5221b^{2}+0.41b,0.5\leq b\leq 3.2,\\ \gamma_{B\left(2\right)}^{2}-6.7153+4.0237b,3.2<b, \end{array}\right\}$$

(29) $$\gamma_{\left(3\right)}^{2}=\gamma_{B\left(3\right)}^{2}-0.3+0.4121b^{2}+0.698b$$

and, similarly, for SMA-based cantilever nanomechanical resonator under compressive stress we get

(30) $$\gamma_{\left(1\right)}^{2}=\left\{\begin{array}{c} \gamma_{B\left(1\right)}^{2}-0.6639b^{2}+1.497\times 10^{-4}b,\;b<0.15,\\ \gamma_{B\left(1\right)}^{2}-0.9057b^{2}+0.1636b,\;0.15\leq b. \end{array}\right\}$$

(31) $$\gamma_{\left(2\right)}^{2}=\gamma_{B\left(2\right)}^{2}-0.086-0.9955b^{2}+0.3883b,$$

(32) $$\gamma_{\left(3\right)}^{2}=\gamma_{B\left(3\right)}^{2}-0.0168-0.6783b^{2}+0.0761b,$$

where *γ*_B(1)_^2^ ≈ 3.52…, *γ*_B(1)_^2^ ≈ 22.03… and *γ*_B(3)_^2^ ≈ 61.70… are the first three consecutive dimensionless flexural resonant frequencies of the SMA-based cantilever nanomechanical resonator without applied internal stress (i.e., *b* = 0). We only note that for suspended nanomechanical resonator the polynomial dependency of the dimensionless resonant frequency on the interlayer stress is given in Ref. [36]. It is evident from Equations (27–32) and results given in [36] that for the SMA-based nanomechanical resonators under tensile / compressive interlayer stress a polynomial dependency of degree two predicts accurately spectrum of the dimensionless flexural resonant frequencies. In addition, for higher vibrational modes impact of stress on resonant frequencies decreases and, in a limiting case of high vibrational modes (i.e., for *n* → ∞), the generated interlayer stress has no impact on resonances, *γ*_(*n*)_ ≈ *γ*_B(*n*)_.

When ultrathin film is sputtered on the resonator surface, it increases the resonator mass and alters the overall flexural rigidity. Importantly, the only unknown parameter in Equations (26–32) is the ultrathin film Young’s modulus, therefore by intentionally changing the elasticity and interlayer stress of SMA-based nanomechanical resonator the Young’s modulus of film can be deduced without necessity of knowing the film density and corresponding unloaded resonant frequencies essentially simplifying experiments. It is worth noting that in contrast the current resonant methods require either experimental estimation of the ultrathin film density prior own film elastic modulus determination [16] or the sophisticated experimental set up / computational tool [12,13,14,15,17]. Plugging Equations (27–32) into Equation (26) and rearranging terms, desired expression enabling calculation of the Young’s modulus of prepared film is given by

(33) $$a_{2}b_{0}^{2}\left(R_{F}\frac{F_{Ta}}{K_{a}}-\frac{F_{Tm}}{K_{m}}\right)+a_{1}b_{0}\left(R_{F}\sqrt{F_{Ta}}-\sqrt{F_{Tm}}\right)+a_{0}\left[R_{F}K_{a}\left(E_{2}\right)-K_{m}(E_{2})\right]=0$$

where *R_F_* = $f_{F\_m}^{\left(n\right)}$/$f_{F\_a}^{\left(n\right)}$, *K_m_*(*E*_2_) = $\sqrt{E_{m}\;r\left(\xi_{m},\eta \right)}$, *K_a_*(*E*_2_) = $\sqrt{E_{a}\;r\left(\xi_{a},\eta \right)}$, *b*_0_ *=* $\sqrt{12L^{2}/\left(WT_{1}^{3}\right)}$ and *a*_1,2,3_ are constants given in Equation (27–32) and/or Ref. [36]. For ultrathin film (i.e., *η <<* 1) term $\sqrt{r\left(\xi_{i},\eta \right)}$ can be further expanded into a Taylor series $\sqrt{r\left(\xi_{i},\eta \right)}\approx 1+\frac{3E_{2}\eta }{2(E_{2}\eta +E_{i})}$, where *i* = *m* and *a*. In general, Equation (33) can be solved only numerically by for instance Bisection or Newton’s method [38]. The analytical solution can be obtained just for the SMA resonator operating under a large tensile stress (e.g., for first two consecutive resonant frequencies of the cantilever resonator *b* > 3.2). In this case, *a*_2_ = 0 and the Young’s modulus of prepared film is then obtained from the following equation

(34) $$E_{2}=\frac{2\left[a_{1}b_{0}\left(\sqrt{F_{Tm}}-R\sqrt{F_{Ta}}\right)-Ra_{0}\sqrt{E_{a}}+1\right]E_{a}E_{m}\eta^{-1}}{\left[2Ra_{0}\sqrt{E_{a}^{3}}+5Ra_{0}E_{m}\sqrt{E_{a}}-2\left(E_{a}+E_{m}\right)\left(a_{1}b_{0}\left(\sqrt{F_{Tm}}-R\sqrt{F_{Ta}}\right)+a_{0}\sqrt{E_{m}}\right)-3a_{0}\sqrt{E_{m}}E_{a}\right]}$$

Shear modulus of sputtered film can be either calculated using a known relationship between the Young’s and shear moduli *G* = *E*/[2(1 + *ν*)] or determined from observed changes in the torsional resonant frequency induced by a phase transformation of NiTi film. The interlayer stress does not affect the dimensionless torsional resonant frequencies λ_(*n*)_. Thus, frequency ratio of the *n*-th torsional vibrational mode of the resonator with SMA in martensite and austenite phases yields

(35) $$\frac{f_{T\_a}^{\left(n\right)}}{f_{T\_m}^{\left(n\right)}}=\sqrt{\frac{G_{a}\;s\left(\zeta_{a},\eta \right)}{G_{m}\;s\left(\zeta_{m},\eta \right)}}$$

where *s*(*ζ_a_*,*η*) = [*ζ_a_*^2^*η*^4^ + 4*ζ_a_η*(1+ 1.5*η* + *η*^2^) + 1]/(1 + *ζ_a_η*), *s*(*ζ_m_*,*η*) = [*ζ_m_*^2^*η*^4^ + 4*ζ_m_η*(1+ 1.5*η* + *η*^2^) + 1]/(1 + *ζ_m_η*), *ζ_m_* = *G*_2_/*G*_m_, *ζ_a_* = *G*_2_/*G*_a_, *G*_2_ and *G*_m_ (*G_a_*) stand for film and resonator shear moduli, respectively. For ultrathin films the higher order terms in *s*(*ζ_a_*,*η*) and *s*(*ζ_m_*,*η*) can be again neglected and, correspondingly, the shear modulus of sputtered film can be calculated from

(36) $$G_{2}=\frac{\left[G_{a}\left(4+6\eta -R_{T}\right)\right]-\sqrt{{\left[G_{a}\left(4+6\eta -R_{T}\right)+G_{m}-G_{m}R_{T}\left(4+6\eta \right)\right]}^{2}-4\left(4+6\eta \right){\left(1-R_{T}\right)}^{2}}}{2\eta \left(4+6\eta \right)\left(R_{T}-1\right)}$$

where *R_T_* = ${\left(f_{T\_a}^{\left(n\right)}/f_{T\_m}^{\left(n\right)}\right)}^{2}\left(G_{m}/G_{a}\right)$.

**Figure 5:**
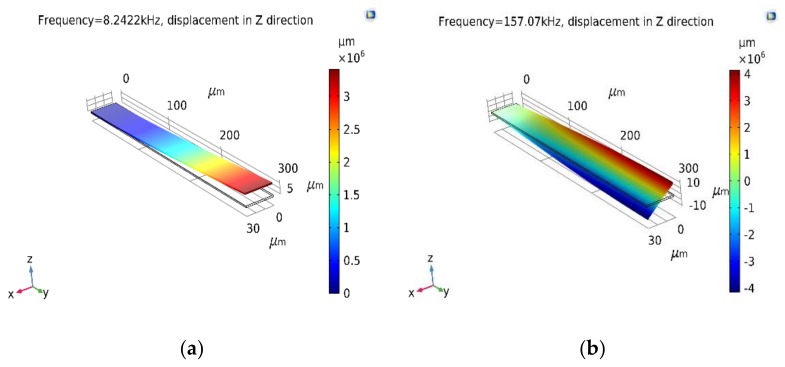
(**a**) Flexural and (**b**) torsional resonant frequencies of the NiTi/Si-based microcantilever in martensite phase with sputtered 50 nm thick NiTi film calculated using COMSOL Multiphysics (details for simulations are given in Supplementary Materials).

**Figure 6:**
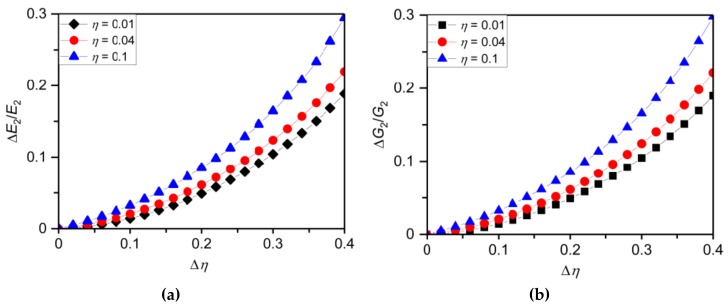
Theoretically achievable relative sensitivity of the NiTi film (**a**) Young’s and (**b**) shear moduli of different thickness ratio *η* = 0.01, 0.04 and 0.1 sputtered on the NiTi/Si resonator of dimensions 300 μm (*L*), 30 μm (*W*) and 1.5 μm (*T*1).

To ensure proposed procedure of the ultrathin film Young’s and shear moduli determination using hybrid SMA (NiTi)-based nanomechanical resonators is practical, the dimensional discrepancies and the uncertainties in generated interlayer stress and resonant frequencies must have a negligibly small impact on the determined elastic properties of investigated ultrathin film. Thickness of film can be measured by ellipsometry with a typical error of sub-nanometer, while the scanning electron microscope with uncertainties ranging from few nm to up to tens of nm are often used to estimate the cantilever length and width. The common temperature fluctuations in experimental device set up have only a minor impact on the SMA (NiTi) film elastic properties and generated interlayer stress [29,30]. Accounting for above uncertainties and measurement errors, we now determine the Young’s and shear moduli of silicon nitride (*E*_2_ = 350 GPa, *G*_2_ = 100 GPa and *ρ*_2_ = 3.2 g/cm^3^) and self-assembled-monolayer (*E*_2_ = 12.9 GPa, *G*_2_ = 5 GPa and *ρ* = 0.675 g/cm^3^) ultrathin films of thickness 20 and 50 nm sputtered on the NiTi/Si cantilever resonator of dimensions *L* = 300 ± 1 μm, *W* = 30 ± 0.5 μm and *T*_1_ = 1.5 ± 0.05 μm, and tension parameter in martensite phase of *b* = −0.1 ± 0.01 that increases with temperature till it reaches value of 0.1 ± 0.012 for an austenite phase. Table 3 gives a comparison of the ultrathin film Young’s and shear moduli calculated using Equations (33) and (36), and determined using numerical simulations performed on COMSOL Multiphysics (see Figure 5) and Supplementary Materials. This comparison confirms that the hybrid SMA (NiTi)-based nanomechanical resonators are indeed suitable for ultrathin film Young’s (shear) modulus determination. Complementary results for theoretically achievable relative sensitivity in determined Young’s and shear moduli of self-assembled-monolayer (SAM) film of different thickness ratios *η* = 0.01, 0.04 and 0.1 sputtered on the identical NiTi-based silicon microcantilever are given in Figure 6. Results given in Figure 6 show that even for a relatively large uncertainty in thickness measurement of 10% the errors in determined Young’s (shear) modulus values are within 5% indicating the enormous potential of NiTi resonators in material testing of ultrathin films.

### 3.3. Mass Spectrometry of Heavy Analytes Using Hybrid SMA (NiTi) Based Nanomechanical Resonator

To begin, we recall a fact that in contrast to a light analyte, where observed shift in the resonant frequency is proportional to the analyte mass and its position of attachment [39], for heavy analyte the resonant frequency shift depends on combination of the analyte mass and stiffness effects, and the position of attachment [40]. The heavy analyte, therefore, cannot be modelled as a point particle making analysis and interpretation of the experimental data challenging. Tamayo et al. [11,41] proposed models that enable predicting the frequency shift with due account for mass and stiffness effects. They proposed that the mass and stiffness effects can be disentangle by either confining the analyte to specific areas of the resonator surface [41] or detecting shifts of multiple vibrational modes [11]. However, analyte confinement in a specific area on the resonator surface and/or analysis of truncation error required in multimode frequency shift measurement limits the real application potential of these two methods [42]. The variable elasticity of the hybrid SMA (NiTi)-based nanomechanical resonators makes possible to overcome limitations of current nanomechanical-based mass spectrometers. Accounting for the theory given in [11] and intentionally changeable properties of NiTi film, the frequency shift of the hybrid SMA-based nanomechanical resonator with an analyte binded at the arbitrary location on the resonator surface can be expressed as

(37) $$\frac{∆f_{F\_i}^{\left(n\right)}}{∆f_{F_{0}\_i}^{\left(n\right)}}=\frac{1}{2}\frac{m_{add}}{m}\theta_{F\left(n\right)}^{2}\left(x_{0}\right)-\frac{3}{2}\vartheta \frac{E_{add}}{E_{i}}\frac{V_{add}}{V_{i}}\kappa_{\left(n\right)}^{2}\left(x_{0}\right)$$

where $∆f_{F\_i}^{\left(n\right)}=f_{F_{0}\_i}^{\left(n\right)}-f_{F\_i}^{\left(n\right)}$, $f_{F\_i}^{\left(n\right)}$ and $f_{F_{0}\_i}^{\left(n\right)}$ are the loaded by analyte and unloaded resonant frequencies of the *n*-th flexural vibrational mode of the SMA nanomechanical resonator, subscript *i* stands for the resonator with SMA film at a given particular value of transformation temperature (e.g., a low temperature martensite phase), *m*_add_, *E*_add_ and *V*_add_ are the analyte mass, stiffness and volume, *x*_0_ is the analyte mass position of attachment (see Figure 1b), *V* is the resonator volume, *κ*_(n)_ is the normalized mode shape curvature and *ϑ* is the dimensionless parameter depending on the analyte shape and contact area [11]. It is evident that Equation (37) enables determination of analyte mass, stiffness and position of attachment from only three measured frequency shifts of a single mode of the hybrid SMA (NiTi)-based nanomechanical resonator with intentionally altered flexural rigidity realized through a variable elasticity of SMA film. As a demonstration, we calculate mass of analyte made of self-assembled-monolayer (i.e., light analyte with a high stiffness-to-mass ratio) and gold (i.e., heavy analyte with a low stiffness-to-mass ratio) bound at *x*_0_ ≈ 0.5 on the NiTi/Si and NiTi/SU-8 hybrid resonators of dimensions 10 μm (*L*), 1 μm (*W*) and 300 nm (*T*_1_), (i.e., NiTi film has a thickness of 100 nm), with and without accounting for the analyte stiffness effect. Three effective Young’s moduli and dimensionless tension parameters needed to calculate mass of analyte are for NiTi/Si resonator *E*_eff_m_ = 79 GPa (*b*_m_ ≈ 1.68), *E*_eff_m/a_ = 99 ± 1 GPa (*b*_m/a_ ≈ 2.12 ± 0.1) and *E*_eff_ a_ = 118 GPa (*b*a ≈ 2.37) and for NiTi/SU-8 they read *E*_eff_m_ = 8.6 GPa (*b*_m_ ≈ 1.61), *E*_eff_m/a_ = 10.2 ± 0.1 GPa (*b*_m/a_ ≈ 1.9 ± 0.08) and *E*_eff_ a_ = 11.4 GPa (*b*_a_ ≈ 2.27), where subscript m/a stands for resonator with a phase transforming SMA film at a given transformation temperature, (i.e., this temperature is higher than the one of SMA in martensite phase but lower than temperature of SMA in austenite phase). Results given in Table 4 show that the high frequency operating hybrid SMA-based nanomechanical resonators can be successfully employed in mass spectrometry of particularly heavy analytes and analytes with a high stiffness-to-mass ratio, where neglecting effect of stiffness yields significant underestimation of determined mass values, (e.g., more than 30% for self-assembled-monolayer). Errors in determined masses are due to accounting for the uncertainties in *E*_eff_m/a_ and *b*_m/a_ caused by thermal fluctuations of a given transformation temperature and variations in NiTi film material properties [26,31,43]. Moreover, interlayer stress exhibits hysteresis, therefore it is required to distinguish whenever experimental data are obtained by heating or cooling of NiTi. In the present case (Table 4), just heating of hybrid SMA (NiTi) resonator is considered.

**Figure 7:**
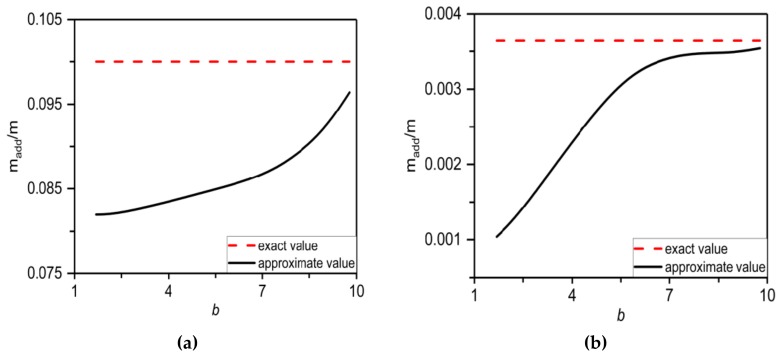
Effect of the interlayer stress represented by a tension parameter *b* on the accuracy of determined mass values using a point mass approximation for (**a**) heavy analyte of *m_add_* = 0.104 and (**b**) light analyte of *m_add_* = 0.0037 located at *x*_0_ ≈ 0.5 on the suspended SMA nanomechanical resonator.

During heating of NiTi not only the resonator effective Young’s modulus increases but also a temperature dependent tensile interlayer stress is generated as discussed above in Section 3.1. In most practical cases, the interlayer stress increases faster than the resonator effective Young’s modulus (see Figure 2, Figure 3 and Figure 4) and, in a limiting case of large interlayer stresses (i.e., σ → ∞), SMA resonator would behave as a string. As such, it can be expected that, alternatively, the mass spectrometry of relatively stiff or heavy analytes can be also performed using hybrid SMA-based nanomechanical resonator with generated high interlayer stress values. In this case, the analyte is modelled as point mass (i.e., detailed theoretical mode of point particle mass can be found in Ref. [6]). Importantly, the numerical computations reveal that the accuracy of determined mass values using a point mass approximation depends strongly on ratio of the analyte mass to the SMA resonator mass. For heavy analyte to determine its mass with a sufficient accuracy requires the extremely high interlayer stress values being generated upon the resonator. For example, Figure 7a shows that for mass ratio *m_add_*/*m* ≈ 0.104 and the SMA resonator in suspended configuration to estimate analyte mass with an error in *m_add_* lower than 5% requires the dimensionless tension parameter *b* > 9.8. On the other hand, the light analyte can be estimated by SMA resonators with moderated to high interlayer stress values. For instance, for *m_add_*/*m* ≈ 0.0037 and the SMA resonator in suspended configuration then the analyte mass can be yet accurately determined even for *b* > 7.3 as illustrated in Figure 7b. In addition, we have also calculated the mass of heavy (Au of *m_add_* ≈ 1.16 pg) and light (SAM of *m_add_* ≈ 40.5 fg) analyte mass located at *x*0 ≈ 0.5 on the suspended NiTi/Si nanomechanical resonator of length 2 μm, width 300 nm and thickness 100 nm. For low interlayer stress values with dimensionless tension parameter *b* ≈ 1.68 the determined mass values using the point mass approximation are for gold 0.921 pg (error in estimated mass of 20%) and for SAM 11.1 fg (error in mass of 73%), while the high interlayer stress of *b* ≈ 9.8 yields the gold mass value of 1.066 pg (deviation in mass of 8%) and SAM mass of 39.4 fg (deviation in mass of 2.7%).

## 4. Conclusions

In summary, we showed that the unique properties of hybrid SMA (NiTi)-based nanomechanical resonators enable essentially improving the achievable resonant frequencies and *Q*-factors and, consequently, to enhance sensitivity of the resonator-based sensors operating in air or viscous fluid. We revealed the enormous capability of these hybrid resonators in nanoscale material characterization by estimating the ultrathin film Young’s and shear moduli from only changes in the resonant frequency of the SMA (NiTi)-based nanomechanical resonator with sputtered film and without requirement of knowing unloaded resonant frequencies as well as density of film and substrate. We showed that the usual discrepancies in dimensions and errors in frequency measurement have a minor impact on the extracted properties. Furthermore, we also found that the SMA-based nanomechanical resonators are capable to determine mass of heavy or stiff analyte from only frequency shift of the single vibrational mode (e.g., fundamental flexural vibrational mode). The explicit analytical formulae for the frequency response of the hybrid SMA-based resonators immersed in viscous fluid are given. Developed procedures of ultrathin film elastic (shear) modulus and heavy analyte mass determination using the hybrid SMA-based nanomechanical resonators are supported by the numerical simulations.

## Figures and Tables

**Table 1:** Theoretically achievable frequencies tuning and *Q*-factor enhancement of NiTi/Si cantilever nanomechanical resonator operating in air and DI water with thickness ratio *η* ≈ 0.17, 0.33 and 0.67.

Measured Quantity	50 nm	100 nm	200 nm
Frequency (in MHz) in air for martensite/austenite	4.32/5.61	4.47/6.28	4.77/7.13
Frequency (in MHz) in DI water for martensite/austenite	2.72/3.66	3.08/4.51	3.66/5.68
*Q*-factor in air for martensite/austenite	153.51/184.09	206.83/262.53	320.71/425.26
*Q*-factor in DI water for martensite/austenite	2.34/2.66	2.77/3.32	3.70/4.66

**Table 2:** Theoretically achievable frequencies tuning and *Q*-factor enhancement of suspended NiTi/Si nanomechanical resonator operating in air and DI water with thickness ratio *η* ≈ 0.17, 0.33 and 0.67.

Measured Quantity	50 nm	100 nm	200 nm
Frequency (in MHz) in air for martensite/austenite	23.38/26.64	22.22/27.29	22.40/30.33
Frequency (in MHz) in DI water for martensite/austenite	18.02/20.54	18.02/22.49	19.34/26.63
*Q*-factor in air for martensite/austenite	500.61/548.65	636.51/735.35	949.01/1173.81
*Q*-factor in DI water for martensite/austenite	5.74/6.15	6.80/7.69	9.30/11.25

**Table 3:** The Young’s (shear) modulus of silicon nitride (Si_3_N_4_) and self-assembled-monolayer (SAM) films of thickness 20 and 50 nm calculated by Equation (33) and Equation (36), and obtained using numerically stimulation.

Measured Quantity	20 nm Si_3_N_4_	50 nm Si_3_N_4_	20 nm SAM	50 nm SAM
Young’s modulus (GPa) by Equation (33)/Simulations	348 ± 12.05/ 355 ± 12.58	353 ± 13.18/ 344 ± 12.47	12.5 ± 0.44/ 12.7 ± 0.43	12.8 ± 0.46/ 12.4 ± 0. 44
Shear modulus (GPa) by Equation (36)/Simulations	97 ± 3.44/ 99 ± 3.41	102 ± 3.68/ 96 ± 3.52	4.8 ± 0.17/ 4.9 ± 0.17	5.1 ± 0.18/ 4.9 ± 0.17

**Table 4:** Determined mass values of self-assembled-monolayer (SAM) of *m_add_* = 5.40 fg and gold (Au) of *m_add_* = 154.4 fg estimated using Equation (37) from only three measured frequency shifts of a fundamental vibrational mode of NiTi/Si and NiTi/SU-8.

Measured Quantity	SAM	Au		
	NiTi/Si	NiTi/SU-8	NiTi/Si	NiTi/SU-8
Analyte mass (fg)/error (%) no stiffness effect	3.28/39.3%	3.66/32.2%	143.1/7.3%	137.6/10.9%
Analyte mass (fg)/error (%) with stiffness effect	5.58/3.3%	5.41/0.2%	158.6/2.7%	153.2/0.5%

## Supplementary Materials

The details of COMSOL modeling are available online at https://www.mdpi.com/1996-1944/12/21/3593/s1.
