# Supplementary material for: Hybrid Shape Memory Alloy-Based Nanomechanical Resonators for Ultrathin Film Elastic Properties Determination and Heavy Mass Spectrometry
Source: Materials (Basel). 2019 Oct 31;12(21):3593. doi: 10.3390/ma12213593 (PMC6862155; doi:10.3390/ma12213593)
Supplement: Supplementary file 1 [file materials-12-03593-s001.pdf]

COMSOL Modeling

We considered there are double layer beam consisting of a substrate with ultrathin film. Therefore, we created a 3D component with two blocks in dimension of L = 300 μm, W = 30 μm, T = 3 μm (see Figure S1) that standing for substrate, and ultrathin film of thickness 20 nm covering above the substrate.

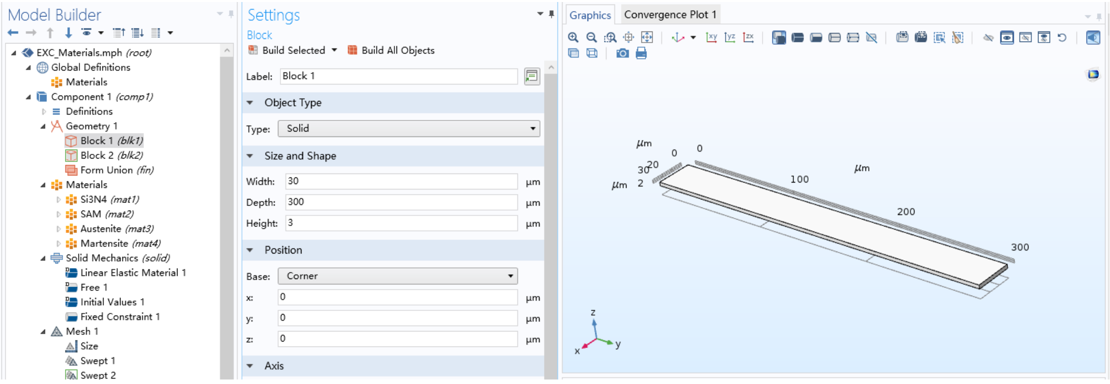

Figure S1. Model of substrate and film

By applying the material properties of substrate martensite phase (E = 30 GPa, ρ = 6.45 g/cm^3, ν = 0.33) and austenite phase (E = 80 GPa, ρ = 6.45 g/cm^3, ν = 0.33), respectively covering above the ultrathin film of SAM (E = 12.9 GPa, ρ = 0.675 g/cm^3, ν = 0.29) or Si_3N_4 (E = 350 GPa, ρ = 3.2 g/cm^3, ν = 0.75). Then, add the solid mechanics physics field to define the linear elastic material and boundary condition, fixing one end or both end for cantilever and bridge configuration, respectively. For mesh part, we chose the swept method and set the maximum size of element 2 μm to find the relative accuracy result (see Figure S2).

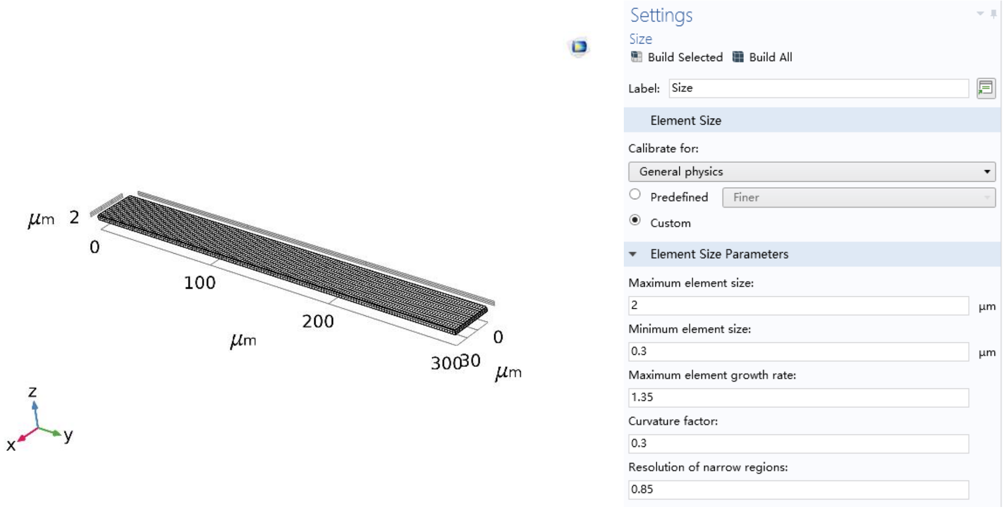

(**a**) (**b**)

Figure S2. The meshing model based on the resonator: (**a**) resonator with mesh and (**b**) mesh setting.

Through computing of the above study, the result of mode shape related to its eigenfrequency can be found that are shown on Figure S3. By checking the mode shape figure we can separate the flexural and torsional resonant frequencies.

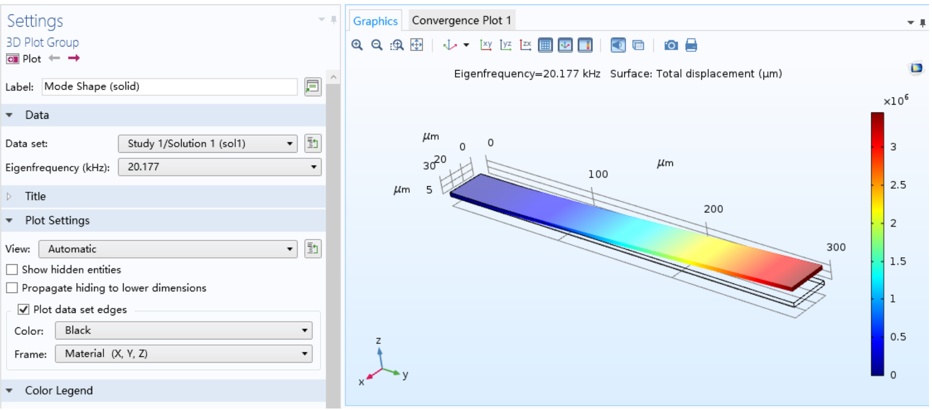

Figure S3. Mode shape for different resonant frequencies.

Through computing of the above study, the result of mode shape related to its eigenfrequency can be found that are shown on Figure S3. By checking the mode shape figure, we can separate the flexural and torsional resonant frequencies. To account for stain and stresses generated by SMA we used approach present in supporting tutorial video of COMSOL https://www.comsol.com/blogs/model-stresses-strains-comsol-multiphysics/
